# Association between perceived social support and induced abortion: A study in maternal health centers in Lima, Peru

**DOI:** 10.1371/journal.pone.0192764

**Published:** 2018-04-12

**Authors:** Luis E. Sánchez-Siancas, Angélica Rodríguez-Medina, Alejandro Piscoya, Antonio Bernabe-Ortiz

**Affiliations:** 1 School of Medicine, Universidad Peruana de Ciencias Aplicadas–UPC, Lima, Peru; 2 Faculty of Epidemiology and Population Health, London School of Hygiene and Tropical Medicine, London, United Kingdom; University of Rochester, UNITED STATES

## Abstract

**Objectives:**

This study aimed to assess the association between perceived social support and induced abortion among young women in Lima, Peru. In addition, prevalence and incidence of induced abortion was estimated.

**Methods/Principal findings:**

A cross-sectional study enrolling women aged 18–25 years from maternal health centers in Southern Lima, Peru, was conducted. Induced abortion was defined as the difference between the total number of pregnancies ended in abortion and the number of spontaneous abortions; whereas perceived social support was assessed using the DUKE-UNC scale. Prevalence and incidence of induced abortion (per 100 person-years risk) was estimated, and the association of interest was evaluated using Poisson regression models with robust variance. A total of 298 women were enrolled, mean age 21.7 (± 2.2) years. Low levels of social support were found in 43.6% (95%CI 38.0%–49.3%), and 17.4% (95%CI: 13.1%– 21.8%) women reported at least one induced abortion. The incidence of induced abortion was 2.37 (95%CI: 1.81–3.11) per 100 person-years risk. The multivariable model showed evidence of the association between low perceived social support and induced abortion (RR = 1.94; 95%CI: 1.14–3.30) after controlling for confounders.

**Conclusions:**

There was evidence of an association between low perceived social support and induced abortion among women aged 18 to 25 years. Incidence of induced abortion was similar or even greater than rates of countries where abortion is legal. Strategies to increase social support and reduce induced abortion rates are needed.

## Introduction

According to the World Health Organization (WHO), induced abortion is defined as the intentional termination of a pregnancy before the fetus can live independently [1]. Worldwide, every year, 35 abortions occurred per 1000 women aged 15 to 44 years, and around 56.3 million women sought for abortion between 2010 and 2014 [2].

In most developing countries, including Peru, abortion is illegal and clandestine; as a result, half of the induced abortions are unsafe, and 98% of the unsafe induced abortions are performed in this kind of countries [3]. Approximately 350,000 induced abortions are performed every year in Peru [4], and about 40% of the unsafe induced abortions are among women aged between 15 and 24 years [5], with a great impact and consequences on health.

Several factors may influence the decision of opting for an induced abortion. These factors include, but are not limited to, age [6], low levels of education [7, 8], marital status [9], age at first sexual intercourse and number of sexual partners [10], low socioeconomic status [7], poor family relationships [11, 12] and lack of social support [13]. Social support, defined as “the perception and authenticity that one is cared for, has assistance available from other people, and that one is part of a supportive social network” [14], is usually associated with a set of inter-personal relationships that facilitate functional and informative support related to person’s health and condition [15, 16].

Several studies have reported that abortion can be stigmatized by relatives and friends, regardless the abortion is legal or not [17–19], suggesting the lack of social support among those women. In Peru, a country where abortion is illegal as in other Latin American countries, there is limited information about the potential impact of social support and familial relationships on induced abortion among young women [11], suggesting that the lack of communication and confidence with the partner/parents were reasons to consider the option of an induced abortion.

Therefore, this study aimed to evaluate the association between perceived social support and induced abortion among young women who were attended in maternal health centers in Southern Lima, Peru. In addition, the prevalence and incidence of induced abortion was estimated.

## Materials and methods

### Study design and setting

Cross-sectional study conducted in three maternal health centers (Ollantay, Manuel Barreto and Daniel Alcides Carrion) located in Southern Lima, part of the Directorate of Health II, in Peru.

### Study population

Potential participants were women aged between 18 and 25 years, attended in at least one of the three aforementioned maternal health centers between March and May 2013. Study subjects were selected in consecutive order after verifying selection criteria. Illiterate women were excluded as questionnaires were self-applied. Incomplete questionnaires, i.e. those without variables of interest (i.e. abortion history and perceived social support), were also excluded from analysis.

### Variables definition

The outcome of interest was the self-report of induced abortion. As induced abortion is a sensitive topic, this variable was calculated indirectly as proposed by Rosier [20], using two questions previously validated in a population-based survey [10]: “Have you ever had a pregnancy that ended in an abortion in the first three months of pregnancy? If so, how many?” and “Did you ever have an abortion that was spontaneous in the first three months of pregnancy? If so, how many?”. The number of induced abortions was estimated from the difference between the number of total abortions and of spontaneous abortions.

The main exposure was perceived social support, assessed using the DUKE-UNC scale, which has been previously validated in Spanish [21]. This tool contains 11 items with responses options ranging from 1 (much less than what I want) to 5 (much as I want) as a Likert scale. The score is calculated by adding up all the items of the tool (range from 11 to 55). Then, that score was split using a cut-off of 32: women with values <32 were categorized as having a low perceived social support, whereas those with score ≥32 were classified as appropriate social support.

Other variables included in the analysis were: maternal health center where women were attended and recruited (Ollantay, Manuel Barreto or Danien Alcides Carrion); age (<22 vs. ≥22 years); education level (< complete secondary, complete secondary, and superior); marital status (ever married vs. single); age of first sexual intercourse (<15 vs. ≥15 years); current number of living children (0–1, 2, 3 or more); and self-report use of contraceptive methods (no/yes).

### Procedures

Initially, the day of visiting each specific maternal health center for participants’ enrollment was randomly selected. Before starting data collection, a screening questionnaire was applied to verify if potential participants accomplished selection criteria. Once a potential participant was identified, the study aims were explained to her, and then, informed consent was read. Questionnaires were self-applied to reduce desirability bias due to the sensitive nature of the questions regarding abortion.

The questionnaire was split in three sections: the first part included demographic characteristics of participants (age, education level, marital status, among others); the second part contained gynecologic and obstetric history as well as the questions regarding abortion; and finally, the third part included the scale of perceived social support. Responding the questionnaire took about 15 to 20 minutes.

### Sample size

The sample size of the study was calculated using Power and Sample Size software (PASS 2008). With a two-sided significance level of 5% and a power of 80% for an association of two binary variables, a total of 270 participants were required to detect an association of 2.5 or more, assuming a prevalence of induced abortion among those with normal social support of 13.6% [10], and a total proportion of women with low social support of 65%. Since questionnaires were self-applied, a 10% of incomplete questionnaires were assumed; therefore, 297 women were needed to be contacted to conduct the study.

### Statistical analysis

Information was double entered using Microsoft Excel for Windows, and then transferred to STATA version 12 (STATA Corp, College Station, TX, US) for analysis.

Initially, population characteristics were compared according to perceived social support and history of induced abortion using the Chi squared test. Then, the prevalence of low perceived social support as well as the prevalence and incidence of induced abortion and their respective 95% confidence intervals (95%CI) were estimated using the exact binomial proportion method. The incidence of induced abortion was calculated by dividing the number of abortions by the number of person-years risk. Because all the women were sexually active, the number of person-years risk was estimated by resting the age of the first sexual intercourse from the age at the moment of the interview as has been reported elsewhere [10].

Finally, generalized linear models, assuming Poisson distribution utilizing the maximum likelihood estimation method, were used to determine the association between perceived social support and induced abortion, controlling for several confounders. Two different models were built to assess the potential impact of covariables in the association of interest. The first one included age, education level, marital status, and maternal health center as covariates; whilst the second one, included the variables of the first model plus current number of living children, age at first sexual intercourse and use of contraceptive methods. Robust standard errors were utilized to estimate relative risks (RR) and 95%CI as well as the population attributable fraction using the *punaf* command in STATA [22]. The population attributable fraction is the proportional reduction in population disease (or event) that would occur if exposure to a risk factor were reduced to an alternative ideal scenario (i.e. absence of exposure) [23].

### Ethical aspects

The study and its informed consent were approved by the Ethical Committee of the Universidad Peruana de Ciencias Aplicadas–UPC, in Lima, Peru. In addition, the study was approved by the directorate of each maternal health center. Verbal informed consent was utilized to guarantee appropriate confidentiality and anonymity.

## Results

A total of 298 women were enrolled in the study, mean age 21.7 (± 2.2) years, 179 (60.1%) had incomplete secondary (<12 years of education), and 118 (39.6%) were single.

### Prevalence of perceived social support

Low perceived social support was present in 43.6% (95%CI: 38.0%– 49.3%) of women. In the bivariable analysis, maternal health center was the only variable associated with low perceived social support. Detailed information regarding the population characteristics according to perceived social support is shown in Table 1.

**Table 1:** Characteristics of the study population by levels of perceived social support.

	Perceived social support		
	Appropriate	Low	p-value*
	(n = 168)	(n = 130)	
***Maternal health center***			
Manuel Barreto	89 (53.0%)	31 (23.9%)	< 0.001
Ollantay	52 (30.9%)	49 (37.7%)	
Daniel Alcides Carrion	27 (16.1%)	50 (38.4%)	
***Age***			
< 22 years	77 (45.8%)	62 (47.7%)	0.75
≥ 22 years	91 (54.2%)	68 (52.3%)	
***Education level***			
< complete secondary	103 (61.3%)	76 (58.5%)	0.14
Complete secondary	25 (14.9%)	12 (9.2%)	
Superior	40 (23.8%)	42 (32.3%)	
***Marital status***			
Ever married	98 (58.3%)	82 (63.1%)	0.41
Single	70 (41.7%)	48 (36.9%)	
***Age at first sexual intercourse***			
< 15 years	101 (60.1%)	90 (69.2%)	0.10
≥ 15 years	67 (39.9%)	40 (30.8%)	
***Current number of living children***			
0–1	100 (59.5%)	74 (56.9%)	0.64
2	49 (29.2%)	44 (33.9%)	
3 or more	19 (11.3%)	12 (9.2%)	
***Use of contraceptive methods***			
No	80 (47.6%)	60 (46.1%)	0.80
Yes	88 (52.4%)	70 (53.9%)	

Results may not add due to missing values.

* P-values were calculated using Chi squared test.

### Life prevalence of induced abortion

Among all women, 17.4% (95%CI: 13.1%– 21.8%) reported had having at least one induced abortion in their life. Among them, 41 (13.7%) reported one induced abortion, 9 (3.0%) reported two, and 2 (0.7%) reported three or more induced abortions. In bivariable analysis, maternal health center, marital status, age at first sexual intercourse, current number of living children, and perceived social support were associated with induced abortion (Table 2).

**Table 2:** Characteristics of the study population by induced abortion.

	Induced abortion		
	No (n = 246)	Yes (n = 52)	p-value*
***Maternal health center***			
Manuel Barreto	113 (94.2%)	7 (5.8%)	< 0.001
Ollantay	75 (74.3%)	26 (25.7%)	
Daniel Alcides Carrion	58 (75.3%)	19 (24.7%)	
***Age***			
< 22 years	119 (85.6%)	20 (14.4%)	0.19
≥ 22 years	127 (79.9%)	32 (20.1%)	
***Education level***			
< complete secondary	141 (78.8%)	38 (21.2%)	0.11
Complete secondary	33 (89.2%)	4 (10.8%)	
Superior	72 (87.8%)	10 (12.2%)	
***Marital status***			
Ever married	157 (87.2%)	23 (12.8%)	0.009
Single	89 (75.4%)	29 (24.6%)	
Age at first sexual intercourse			
< 15 years	144 (75.4%)	47 (24.6%)	< 0.001
≥ 15 years	102 (95.3%)	5 (4.7%)	
***Current number of living children***			
0–1	147 (84.8%)	27 (15.5%)	0.004
2	80 (86.0%)	13 (14.0%)	
3 or more	19 (61.3%)	12 (38.7%)	
***Use of contraceptive methods***			
No	117 (83.6%)	23 (16.4%)	0.66
Yes	129 (81.7%)	29 (18.4%)	
***Perceived social support***			
Appropriate	150 (89.3%)	18 (10.7%)	< 0.001
Low	96 (73.8%)	34 (26.2%)	

Percentages have been calculated by rows.

* P-values were calculated using Chi squared test.

### Incidence of induced abortion and its association with perceived social support

**Fig 1:**
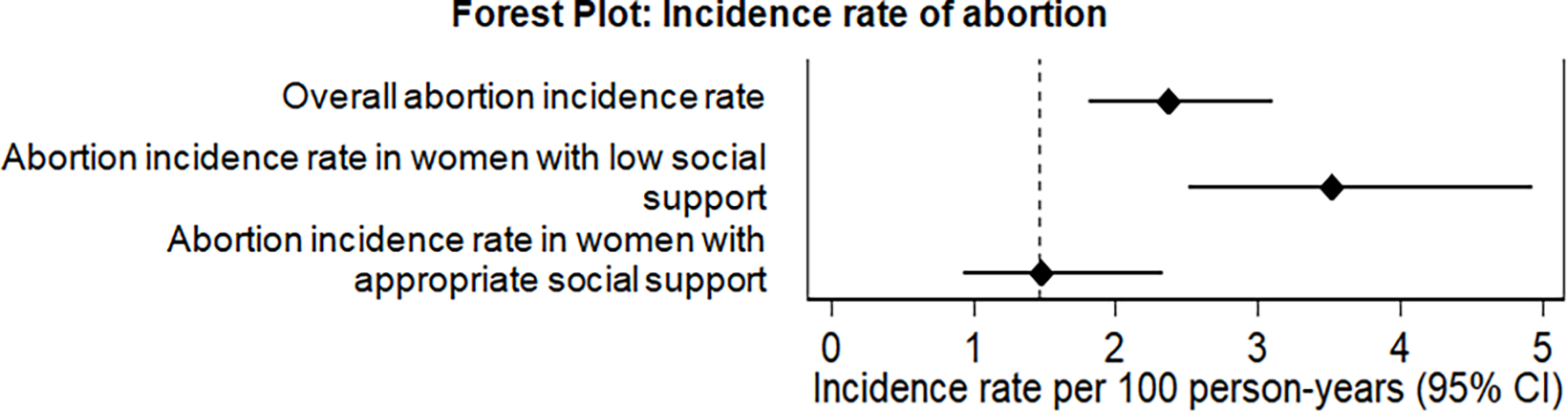
Incidence rate of abortion: Overall and by perceived social support. For comparison, the dashed vertical line indicates the rate incidence of induced abortion in countries where abortion is legal (USA, 2014 [24]).

The incidence of induced abortion was 2.37 (95%CI: 1.81–3.11) per 100 person-years risk (Fig 1). Women with appropriate social support had lower incidence of induced abortion (1.47; 95%CI: 0.93–2.33) when compared to women with low social support (3.52; 95%CI: 2.51–4.93).

In multivariable model, there was evidence of an association between low perceived social support and induced abortion (RR = 1.94; 95%CI: 1.14–3.30) after controlling for age, education level, marital status, maternal health center, current number of living children, age at first sexual intercourse, and current use of contraceptives (S1 Table). Accordingly, the population attributable fraction due to low social support was 31.7% (95%CI: 4.4% - 51.2%).

## Discussion

### Main findings

Our results show strong evidence of the association between perceived social support and induced abortion: low social support was associated with a twice increase of the incidence of induced abortion after controlling for potential confounders. Moreover, the population attributable fraction shows that increasing social support in this population would reduce in almost a third the number of cases of induced abortion. Additionally, almost a fifth of women aged between 18 and 25 years reported had having at least one induced abortion, a country where abortion is illegal and clandestine, whilst more than 40% of participants reported low levels of social support.

### Perceived social support and induced abortion

Some manuscripts have reported that social support may influence the development of positive behaviors towards health and self-esteem, including the reduction of stress [25, 26]. Moreover, greater levels of social support improve quality of life as well as promoting physical and psychological well-being in women [27, 28], but also in the product. For example, having a supportive partner may reduce the experience of low birth weight in the product or have a pregnancy loss [29]. On the contrary, low levels of social support predispose to higher risk of disease, increase other adverse results and mortality [30, 31].

The results of our study are similar to previous reports evaluating the impact of social support on other diseases and other life events [27, 31], suggesting that appropriate social support is needed, even before pregnancy, to reduce the possibility of abortion. For example, a previous study found that greater perceived social support from relatives, partner and friends, was related to increased feelings of self-efficacy for coping with abortion [32]. Despite of being illegal and its potential negative consequences, inducing an abortion depends on different factors, with social support playing perhaps an important role. Therefore, further studies are needed to better understand how the decision of inducing an abortion is taken, with the ultimate goal of guarantying an appropriate social support.

### Incidence of induced abortion

Rates of induced abortion found in this study are relatively high: an incidence of 2.4 per 100 person-years of risk in women aged 18 to 25 years is comparable to the annual rate of United Kingdom [33] and United States [34, 35]. As abortion is illegal, it is difficult to appropriately determine the real incidence of induced abortion in Peruvian settings. However, comparisons with other studies conducted in our context are possible. For example, Ferrando found an incidence of induced abortion of 5.2% among women aged between 15 and 49 years using clinical reports of abortion complications [4]. To compare this figure with our result, we applied an adjustment factor based on the proportion of women that are aged between 18 and 25 years from the total of women of 15 to 49 years (i.e. 40% according to the results of the 2007 census [36]. Thus, an adjusted incidence of 5.9% per year was obtained, which is higher than the incidence reported in the aforementioned study, but similar to the estimate of 6.1% reported in a population-based survey among women between 18 and 29 years [10]. Our findings, therefore, suggest that countries like Peru, where the abortion is illegal, may have at least the same (or even higher) abortion rates than countries where it is legal and safe [3, 24].

### Low levels of perceived social support

About 40% of women enrolled in the study reported low levels of social support. Many reasons might explain these findings. For example, it is well known that family and relatives, especially the support of the father and partner, are important during the period of pregnancy and children growth [12, 17]. In addition, education level and socioeconomic status seem to be robustly associated with the perception of social support [8, 13], that can be the case as the women enrolled in this study lived in poor areas in Lima. Surprisingly, a previous study reported that women with unwanted pregnancy were demographically similar to those with wanted pregnancies, although the difference was focused on the psychological profile and maternal characteristics [37]. Thus, our study supports the need of a deep assessment of this kind of populations to appropriately suggest interventions to reduce induced abortion and increase social support. For example, the access to contraceptive methods may be limited if social support, mainly for their partners, is not present [38].

### Public health relevance

There is a clear relationship between social support and incident cases of induced abortion as previously explained. Interventions need to focus on building an appropriate social support to reduce rates of induced abortion. However and most importantly, our results highlight the burden of induced abortions in resource-constrained settings such as Peru. According to that, there are some points that need to be pointed out. First, most induced abortions are clandestine and unsafe, which can have a great impact in women’s health and safety, as well as huge costs to a resource-constrained health system. Second, the high rates of induced abortion found in this study emphasize the lack of appropriate sexual education as well as the failure of adequately use of contraceptive methods. For example, previous studies have demonstrated that less than half of women in reproductive age reported using a contraceptive method during sexual intercourses [10]. Third, our finding underscores the high number of unwanted pregnancies in our study population. A previous study reported that a third of children were unwanted [4] and part of them were ended as abortions. Based on this, there is a need of improving sexual education, information and access to contraceptive methods, in addition to improve social support, to prevent clandestine and unsafe induced abortions.

### Strengths and limitations

This study is one of the few reporting data regarding the prevalence and incidence of induced abortion in a country where it is illegal. However, this study has several limitations. First, the cross-sectional nature of the study design prevents us to determine directionality as reverse causality can be present. Second, although self-applied questionnaires were used, all studies about induced abortion have underreporting due to social desirability bias as well as potential stigmatization related to the legal concerns as this procedure is restricted. In addition, some recall bias might arise as several years might have lapsed between the event and the interview. However, we tried to reduce this bias by enrolling young women aged between 18 and 25 years. Third, a non probabilistic sampling technique was used to recruit participants; as a consequence, results might not be inferable to the general population. In addition, illiterate women were excluded from the study and this decision might have an impact on our estimates. Nevertheless, our findings are similar to previous studies conducted in our context [4, 10]. Fourth, the DUKE-UNC scale assesses perceived and not real social support, and this might affect our estimates. Finally, some confounders, especially socioeconomic status, were not assessed and hence, not included in the regression models with potential impact on our results. Nonetheless, our models were controlled by education level, a proxy of socioeconomic status [39]. As induced abortion might be present in all the socioeconomic groups, the potential effect of this variable may be small.

### Conclusions

In conclusion, there was evidence of an association between low perceived social support and induced abortion among women aged between 18 and 25 years. The incidence of induced abortion was similar, o even greater, than rates of countries where abortion is legal. A great proportion of women reported low levels of social support. Appropriate strategies are needed to increase social support and reduce induced abortion rates in young women.

## Supporting information

S1 Table Association between perceived social support and induced abortion: Crude and adjusted models. (DOC)
