# Supplementary material for: Association between perceived social support and induced abortion: A study in maternal health centers in Lima, Peru
Source: PLoS One. 2018 Apr 12;13(4):e0192764. doi: 10.1371/journal.pone.0192764 (PMC5896907; doi:10.1371/journal.pone.0192764)
Supplement: S1 Table — (DOC) [file pone.0192764.s001.doc]

**S1 Table:** Association between perceived social support and induced abortion: crude and adjusted models

|  | **Crude model** | **Adjusted model 1*** | **Adjusted model 2**** |
| --- | --- | --- | --- |
|  | **RR (IC95%)** | **RR (IC95%)** | **RR (IC95%)** |
| ***Perceived social support*** |  |  |  |
| Appropriate | 1 (Reference) | 1 (Reference) | 1 (Reference) |
| Low | 2.44 (1.44 – 4.12) | 1.90 (1.07 – 3.37) | 1.94 (1.14 – 3.30) |

RR = relative risk; 95%CI = 95% confidence intervals

* Adjusted by age, education level, marital status and maternal health center.

** Adjusted by age, education level, marital status and maternal health center, current number of living children, age at first sexual intercourse, and use of contraceptive methods.
